# Demodulation of Fibre Bragg Grating Sensors by Using Cumulative Sum as a Preprocessing Method

**DOI:** 10.3390/s25030634

**Published:** 2025-01-22

**Authors:** Sławomir Cięszczyk, Marek Kida, Patryk Panas

**Affiliations:** Institute of Electronics and Information Technology, Lublin University of Technology, Nadbystrzycka 38A, 20-618 Lublin, Poland; m.kida@pollub.pl (M.K.); p.panas@pollub.pl (P.P.)

**Keywords:** FBG sensors, demodulation algorithm, wavelength shift

## Abstract

Fibre Bragg gratings are one of the most popular sensors with a huge number of applications. Their most important advantage is signal modulation consisting in shifting the spectrum in the wavelength domain. Determining the wavelength shift is the most important issue in precise measurements of various quantities. New demodulation methods are constantly being developed. Many of them have good properties, but they do not gain much polarity. This is partly due to their high complexity and partly to a small improvement in the accuracy of determining the wavelength shift in relation to classical methods. Cumulative preprocessing is a very simple method of spectrum processing with the property of reducing the influence of noise on the result. The method can be used directly or with additional algorithms. In this article, we demonstrate the advantages of this method and the possibilities of combining it with other signal processing methods. We show that this method is much simpler than the spectrum denoising methods and additionally simplifies the next stage of the algorithm, i.e., determining the wavelength shift itself.

## 1. Introduction

Fibre Bragg gratings are periodic structures with a significant number of sensor applications. Such gratings have all the positive properties of fibre optics, such as small size or resistance to electromagnetic radiation and harsh environments. The main advantage of periodic structures is the encoding of information in wavelengths. A physical quantity such as temperature or strain affecting the sensor element causes a shift in the optical spectrum without distorting it. The most popular is a grating with a Gaussian shape of the optical spectrum. Depending on the internal structure, the shape of the spectrum will change. To determine this shift, appropriate interrogation systems or general-purpose spectrum analysers are used. In the case of spectrum measurements, the next step is to use algorithmic procedures for the precise determination of the wavelength shift. The basic problem in achieving a good result is the level of the signal-to-noise ratio. In the case of dedicated interrogators, it may also be important to perform spectral measurements with high resolution. Newly proposed FBG demodulation methods use increasingly advanced algorithms, including those that use artificial intelligence [1,2,3]. However, the problem here is still developing simple and efficient algorithms that would gain recognition among the scientific and engineering community and enter into widespread use.

Research on demodulation algorithms can be divided into three main parts. The first group is searching for better methods of spectrum denoising. The second group is identifying the simplest methods, in particular with low computational complexity. Finally, the third group is identifying the methods with the best metrological parameters regardless of computational complexity. Among the methods that minimise the impact of noise on demodulation, algorithms such as empirical mode decomposition [4], improved wavelet transform [5], wavelet packet decomposition [6] or Dynamic Statistical Threshold Detection algorithm [7,8] are used. In some FBG applications, in addition to noise, spectral distortions may appear, which may additionally affect the quality of determining the wavelength shift depending on the algorithm used [9]. Recently, scientific publications have also discussed issues related to the simultaneous demodulation of several FBGs [10,11]. Among them, there are also two sensors with mutually overlapping spectra [12,13]. For the simultaneous interrogation of many sensors, simple algorithms with low numerical complexity are preferred. Such solutions include special methods of implementing the second-order polynomial [14] with its additional simplification in order to reduce the computation time [15]. Simple and effective algorithms also include the three-point estimator [16].

The theoretical background of the proposed cumulative spectrum processing method will be presented in the following part of the article. Next, a comparison of several versions of the new algorithm will be presented and their evaluation with the commonly used cross-correlation method and Hilbert transform for simulation data. In the next step, the application of the algorithms for experimental data will be compared. Development and specification of the algorithm properties consisted of the following steps:

- generation of data for testing the algorithms,
- development of the basic cumulative sum algorithm with logistic and linear approximation,
- development of reference algorithms: Hilbert transformation and cross-correlation with Hilbert transformation, testing and comparing the developed algorithms with the reference algorithms,
- modification of the algorithms: wavelet filtration, calculation of the square value of the basic spectrum, difference in cumulative spectra calculated from left to right and from right to left,
- testing of the modified algorithms,
- analysis of experimental data.

## 2. Analysis of Selected Demodulation Algorithms

The Bragg wavelength of gratings depends on the effective refractive index of the core *n_eff_* and grating period *Λ*:

(1) $$\lambda_{B}=2\cdot n_{eff}\cdot \Lambda .$$

The simplest model of the spectrum is the Gaussian shape, where the reflection spectrum can be written as follows:

(2) $$R\left(\lambda \right)=R_{0}exp\left(-4ln2{\left(\frac{\lambda -\lambda_{B}}{∆\lambda_{B}}\right)}^{2}\right),$$

where $R_{0}$ is the peak amplitude and $∆\lambda_{B}$ is its full width at half maximum. The Gaussian wavelength is achieved by using appropriate apodisation techniques, which are designed to reduce the intensity of the side lobes.

The cumulative curve method can be classified as a method that transforms the spectrum before calculating the shift. Such methods include the Hilbert transformation and the differential filter. The Hilbert transformation can be written as follows:

(3) $$\hat{R}\left(\lambda \right)=\frac{1}{\pi }p.v.\int_{-\infty }^{+\infty }R\left(\lambda \right)\frac{1}{\lambda -\tau }d\tau .$$

The basic observed change in the spectrum is the transformation of the resonance peak into a curve passing through the zero value. The Hilbert transformation can be preceded by calculating the cross-correlation function between the reference spectrum $R_{0}$ and the analysed spectrum $R_{1}$:

(4) $$C_{j}=\sum_{i=0}^{N-1}R_{0}\left(\lambda_{i}\right)R_{1}\left(\lambda_{i+j}\right),$$

for *j* = 0, 1, 2, …, (2*N* − 1). This method is referred to as the zero-crossing algorithm [17]. An additional process can be the use of low-pass FIR filters [18].

Another proposed transformation of the optical spectrum is the determination of the first derivative after the Hilbert transformation. Such an algorithm is used to distinguish several peaks of gratings recorded on one optical fibre [19]. The most popular methods using signal transformation include the fast phase correlation method [20].

The cumulative sum method is difficult to classify directly into one selected group of methods [21,22]. The spectrum transformation itself can be classified as a broadly understood filtration method. This group includes all preliminary denoising methods, the Hilbert transformation and the linear phase operator [23]. However, this operator is a differentiating filter with a smoothing component. Such methods operate in the wavelength domain of the measured spectrum without using parameters after transformation to another (Fourier) domain. In the second step of the algorithm, it is necessary to match the appropriate function to the modified spectrum. After the cumulative filtration, approximation with a linear curve or a logistic function can be used.

The cumulative sum of points of measured spectra can be written as:

(5) $$A\left(\lambda_{i}\right)=\sum_{i=0}^{i}R_{0}\left(\lambda_{j}\right).$$

From the perspective of digital signal processing, such an operation can be performed using an integrator, whose difference equation can be written as follows:

(6) $$y\left[n\right]=\sum_{k=0}^{n}x[k],$$

where *x*[*k*] is input signal and *y*[*n*] is output signals. Alternatively, integrator with a feedback form, i.e., as a recursive system:

(7) $$y\left[n\right]=x\left[n\right]+y[n-1].$$

**Figure 1:**
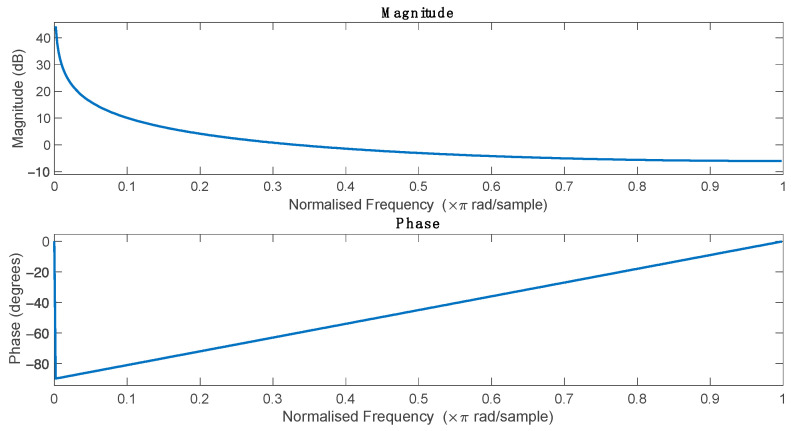
Magnitude and phase response of discrete-time integrators.

The impulse response of such a filter is a unit step, and in the frequency domain it is a low-pass filter (Figure 1). This also means that such a filter significantly attenuates higher frequencies and thus, attenuates noise. It should also be noted that this is linear phase filter meaning that it maintains a constant delay for all frequencies.

**Figure 2:**
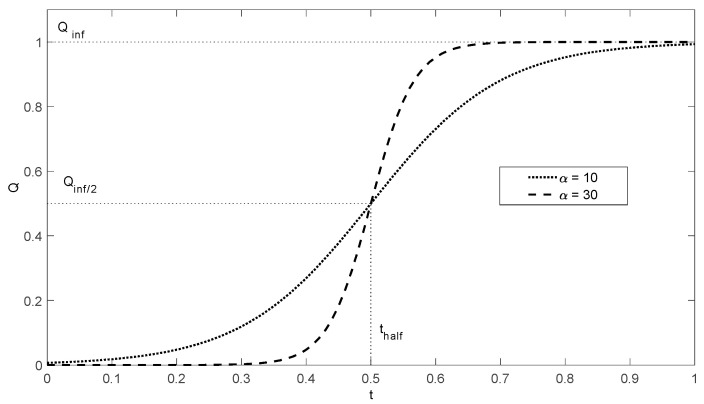
Graphic illustration of model logistic curves.

The cumulative spectrum method with additional preliminary smoothing filtering is used for spectral analysis in UV spectroscopy [24]. However, it is used is to improve the analytical signal and not to determine the spectral shift. After cumulative processing of the spectrum, the resulting curve can be approximated by a logistic curve. This curve is described by three parameters:

(8) $$N\left(t\right)=\frac{M}{1+ce^{-at}},$$

or in an equivalent form:

(9) $$N\left(t\right)=\frac{M}{1+e^{-r(t-t_{0})}},$$

if $c=exp\left(rt_{0}\right)$, *M*—maximum value of the function, *r*—rate of growth, $t_{0}$ is the point with the highest rate of growth of the curve and is located at the half of the maximum height of the function. The graphical representation of the logistic function is shown in Figure 2.

**Figure 3:**
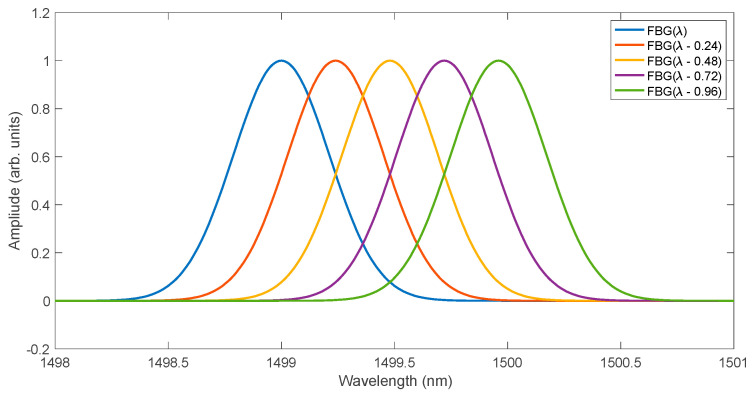
Example FBG spectra with a wavelength shift.

In a typical application of the logistic curve, we are interested in two of its parameters, namely the growth rate and the $t_{0}$ point. In the case of FBG spectra analysis, only the inflexion point is important because the growth rate of the function does not change. Additionally, since the cumulative FBG spectrum is a symmetric and odd function with respect to the inflexion point, the shift in this curve can only be approximated in the range in which its significant growth occurs. Such approximation can also be performed with a linear function. Figure 3 shows five Gaussian shape spectra of a Bragg grating with full width at half maximum of 0.5 nm. Subsequent spectra differ from each other by a shift of 0.24 nm. A spectral resolution of 0.01 nm was assumed. Such spectral parameters allow for explaining the principle of operation of the algorithm consisting in summing subsequent spectrum samples.

**Figure 4:**
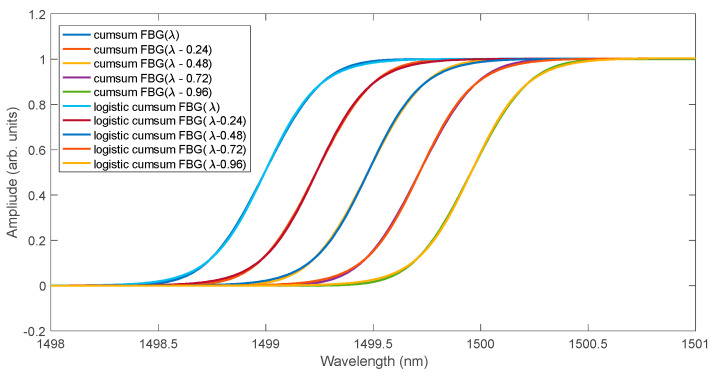
Cumulative sum of FBG spectra calculated from the spectra of Figure 3 together with their approximations using logistic curves.

**Figure 5:**
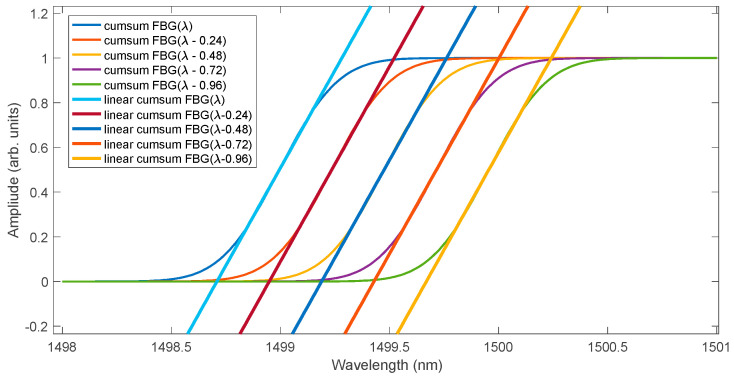
Approximation of slopes of cumulative curves using a linear function.

The shape of the spectrum after the integrating filter is shown in Figure 4. At the same time, the cumulative curves were approximated using a logistic function. The fit of the cumulative spectra and their logistic approximation is very good in the entire range. In the range of the greatest increase in the value of the cumulative functions, only their shift occurs for individual spectra. In this range of the cumulative spectrum, instead of the logistic function, approximation using a linear function can be used. The determined linear function will shift similarly to the logistic function (Figure 5).

**Figure 6:**
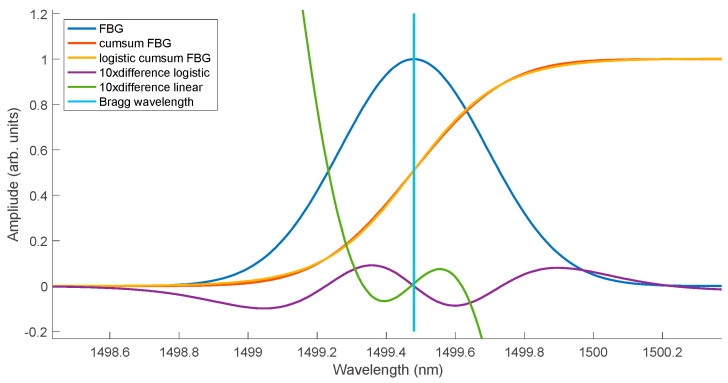
Difference between the cumulative curve and its approximation using a logistic curve and a linear function.

Figure 6 shows the quality of the fit of the logistic function and the linear function to the cumulative spectrum. For better visualisation, a single FBG spectrum and its cumulative version normalised to unity are shown. The differences between the cumulative spectrum and its approximations are magnified tenfold. The errors for both types of approximations pass through zero at the Bragg wavelength. The maximum approximation error using the logistic function does not exceed 1% in the entire wavelength range. The approximation error using the linear function grows very quickly outside the full width at half maximum of the Gaussian curve.

**Figure 7:**
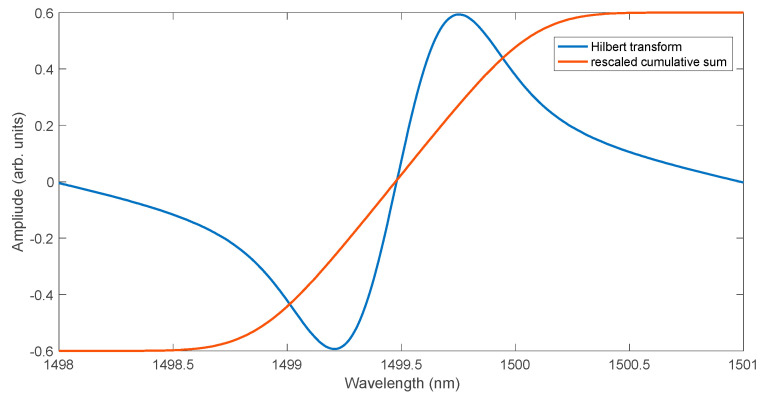
Comparison of the rescaled cumulative curve with the Hilbert transformation of the FBG spectrum.

**Figure 8:**
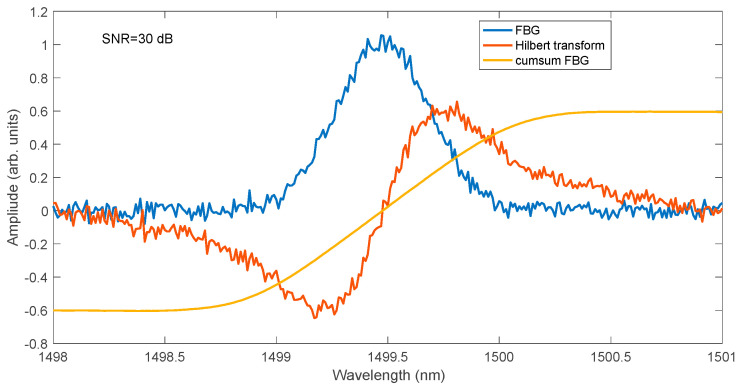
FBG spectrum and comparison of the rescaled cumulative curve with the Hilbert transformation of the FBG spectrum.

In processing the spectra of Bragg gratings, the Hilbert transform can be used. It can be applied directly to the measured FBG spectrum or, with better results, to the calculated cross-correlation function. A comparison of the Hilbert transform of the modelled FBG spectrum with the scaled values of the cumulative spectrum is shown in Figure 7. The Hilbert transform is a global transform, i.e., each individual value on the wavelength axis is determined using all values of the spectrum subjected to the transformation. In the case of the cumulative sum, the values of all previous points of the processed spectrum are necessary to calculate the value of each point on the wavelength axis. The method of calculating the cumulative curve as an integral filtering has strong properties of attenuating higher frequencies. The effect of signal smoothing can be observed for the FBG spectrum model for 30 dB SNR shown in Figure 8. The noise is visible both for the basic FBG spectrum and for its Hilbert transform. The scaled cumulative curve normalised to the Hilbert transform level is, however, smooth.

## 3. Properties of the Cumulative Sum Algorithm and Its Modifications

To assess the properties of the proposed method for determining the Bragg wavelength shift, simulations of spectra were performed for a 0.5 nm wide grating model for 1001 Bragg wavelength shift values in the range from 0 to 1 nm. A spectral resolution of 0.01 nm was assumed. Simulations were performed for seven noise levels from 30 to 60 dB. The entire simulation set therefore included 7007 spectra. As a metric for comparing the accuracy of determining the spectral shift for individual algorithms, we used the root mean square error (RMSE) from M simulation spectra:

(10) $$RMSE=\sqrt{\frac{\sum_{m=1}^{M}{\left(∆\lambda_{t}-∆\lambda_{e}\right)}^{2}}{M}},$$

where $∆\lambda_{t}$ is the actual (given) wavelength shift and $∆\lambda_{e}$ is the wavelength shift estimated by the specified algorithm.

**Figure 9:**
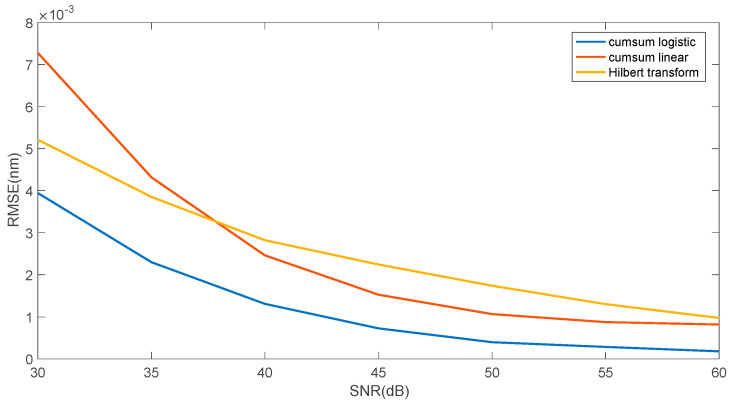
Comparison of the root mean square error for three algorithms: cumulative sum—linear approximation, cumulative sum—logistic approximation, Hilbert transform—zero crossing.

**Figure 10:**
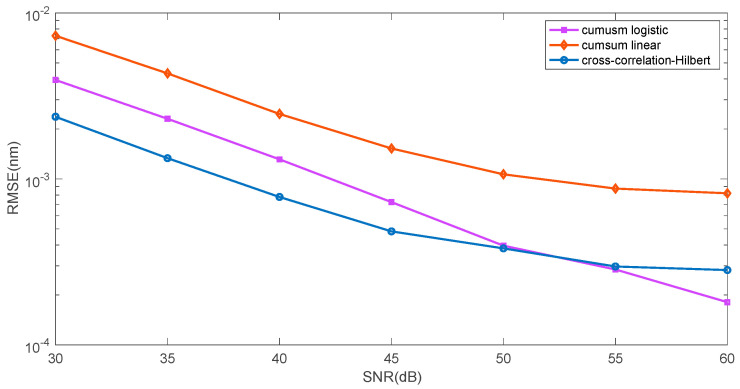
Comparison of root mean squared error for three algorithms: cumulative function—linear approximation, cumulative function—logistic approximation, cross-correlation—Hilbert transform—zero crossing.

Figure 9 presents a comparison of the two proposed approximation methods (linear and logistic function) and the Hilbert transformation zero crossing determination method. Figure 10 shows the mean square error value for determining the wavelength shift on a logarithmic scale. The figure shows the two proposed methods, and a three-step method commonly used for shift estimation, which can be considered a reference method. This method consists of performing a Hilbert transformation from the cross-correlation function between the reference spectrum and the analysed spectrum. Then, the zero position is determined from the Hilbert transform using a linear approximation between its minimum and maximum value. The error in determining the shift is smaller for the reference method for smaller SNR values in the range of 30–45 dB. For larger SNR values, the logistic function approximation method is better.

**Figure 11:**
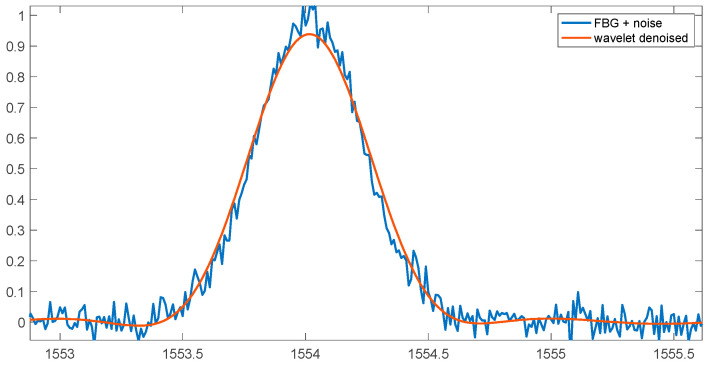
FBG spectrum with 30 dB noise and wavelet-denoised spectrum.

Before the actual analysis of the Bragg wave shift, additional denoising can be applied. One of the better methods in this field is the wavelet transform. Figure 11 shows the noisy spectrum and the denoised spectrum using the wavelet transform. The analysis method using the Symlet wavelet of the sixth order was used. The denoised spectrum contains the sum of the fifth and sixth level of decomposition.

**Figure 12:**
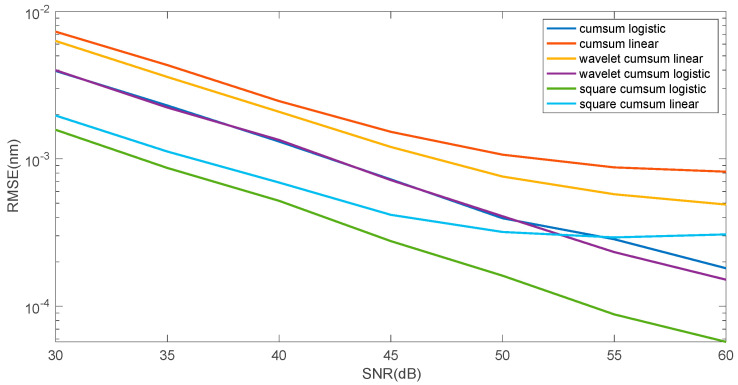
Comparison of the properties of the Bragg wavelength shift determination algorithms in the case of using wavelet filtering and squaring the cumulative signal amplitude.

Figure 12 shows that denoising has a small effect on the error of the linear function approximation method and does not change the logistic function approximation error at all. This is due to the good denoising properties of the cumulative sum method itself and the additional averaging, which is the fit of both the logistic and linear models to the cumulative curve. However, a reduction in the mean square error can be achieved by squaring the individual points of spectrum before the cumulative sum calculation.

**Figure 13:**
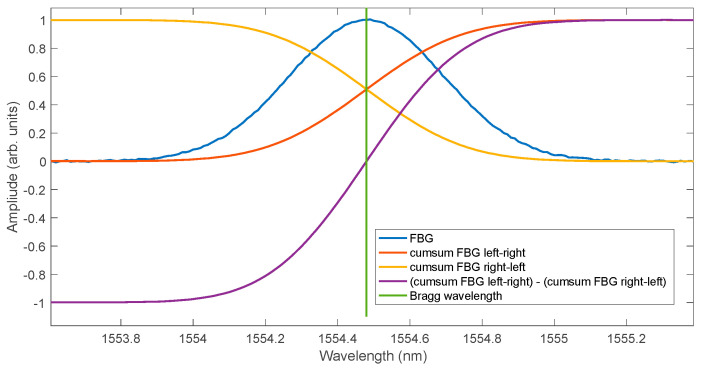
Normalised cumulative spectra calculated from left to right and from right to left.

**Figure 14:**
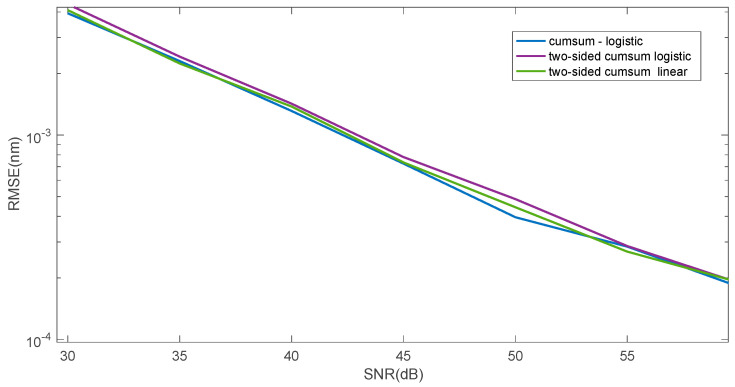
Comparison of the properties of the Bragg wavelength shift determination algorithms using right- and left-sided cumulative sum.

The cumulative sum method can also be modified in a way other than wavelet filtering. The summation of spectrum samples can also be performed from the side of larger wavelength values. In the next step, subtraction can be performed between the cumulative sum calculated from the left and right side. As a result, the curve has odd symmetry with respect to the point whose coordinates have the value of the Bragg wavelength on the wavelength axis and zero on the amplitude axis (Figure 13). The most important advantage of such a modification of the algorithm is obtaining the mean square error of the linear approximation of the difference curve at the level of the error of the approximation with the logistic function of a single cumulative curve (Figure 14). However, for the difference in cumulative sum, the use of linear and logistic approximation does not change the level of the obtained root mean square error.

**Figure 15:**
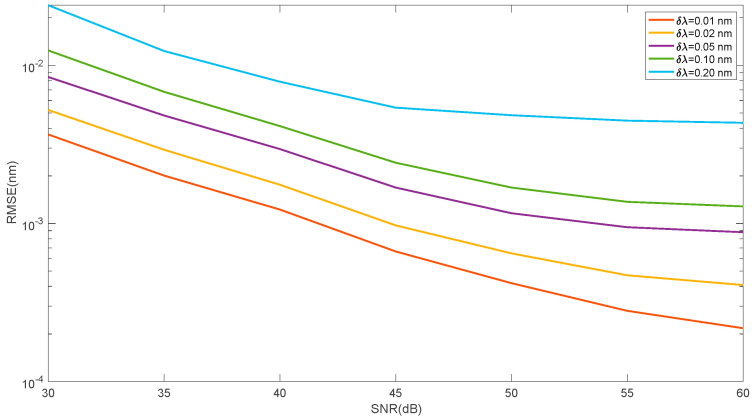
Root mean square error of Bragg wavelength shift determination for different values of spectrum resolution for the left- and right-side cumulative sum subtraction method.

The higher resolution of the spectrum measurement means more points with information about the shape of the Bragg grating spectrum and its position. Reducing the resolution causes an increase in the root mean square error of the Bragg wavelength estimation. Figure 15 shows the dependence of the mean square error on the signal-to-noise ratio for five resolution values. The comparison concerns the linear approximation method for the left-side and right-side difference in the cumulative sum.

**Figure 16:**
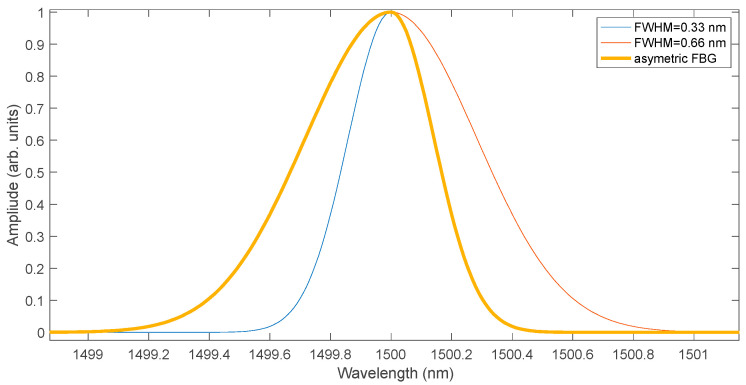
Asymmetric FBG spectrum as a composite of two symmetric spectra, one with twice the width of the other.

As a result of the FBG manufacturing process, deviations of their spectra from Gaussian-like symmetric curves may occur. Distortions also appear during measurements due to stress inhomogeneity along the grating length. In order to verify the properties of the cumulative sum algorithm for asymmetric spectra, a modification of the generated data were performed. The spectrum model used for the analysis of distorted FBG signals [9] is based on the combination of two Gaussian shapes with different half-widths [25]:

(11) $$R\left(\lambda \right)=\left\{\begin{array}{c} R_{0}exp\left(-4ln2{\left(\frac{\lambda -\lambda_{B}}{∆\lambda_{B}}\right)}^{2}\right),\lambda <\lambda_{B}\;\\ R_{0}exp\left(-4ln2{\left(\frac{\lambda -\lambda_{B}}{\chi \cdot ∆\lambda_{B}}\right)}^{2}\right),\lambda \geq \lambda_{B} \end{array}\right.$$

where *χ* is responsible for the degree of asymmetry. The shape used to model asymmetric spectra and their shift under the influence of the measured quantity is shown in Figure 16. The figure contains an asymmetric spectrum and two symmetric spectra with FWHM of 0.33 nm and 0.66 nm, respectively. The left half of the asymmetric spectrum has a symmetric spectrum shape with a FWHM of 0.66 nm while the right half has a symmetric spectrum shape with a FWHM of 0.33 nm. The resulting FWHM value of the asymmetric spectrum is 0.5 nm.

**Figure 17:**
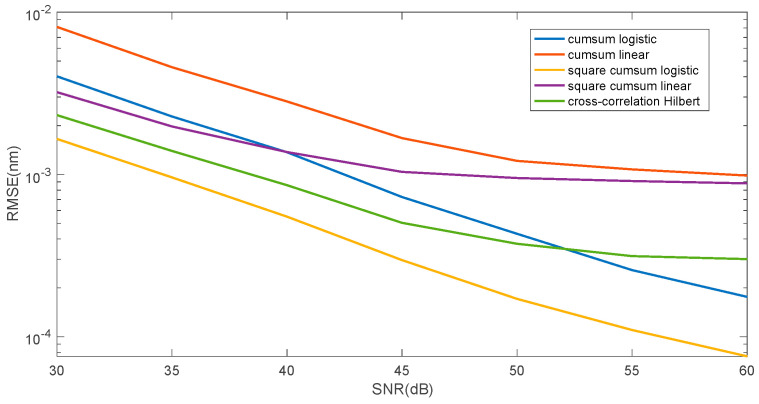
Mean square error in determining the asymmetric shift in the FBG spectrum for five algorithms: cumulative function—linear approximation, cumulative function—logistic approximation, spectrum square—cumulative function—linear approximation, spectrum square—cumulative function—logistic approximation, cross-correlation—Hilbert transform—zero crossing.

It follows from Figure 17 that the distortion from the symmetry of the spectrum does not affect the deterioration of the accuracy of the demodulation of its shift. The smallest mean square error is characterised by the logistic approximation algorithm of the cumulative sum calculated from the square value of the spectrum intensity. This method is better than the algorithm for calculating the Hilbert transform from cross-correlation.

In principle, this comparison of methods can only be based on the comparison of RMSE plots for different SNR values. Therefore, the classification of the developed methods together with the reference methods can be presented in the following order:

(a) Hilbert transform—linear approximation,
(b) cumulative sum—linear approximation,
(c) wavelet—cumulative sum—linear approximation,
(d) cumulative sum—logistic approximation,
(e) difference in two-sided cumulative sum—linear approximation,
(f) wavelet—cumulative sum—logistic approximation,
(g) cross-correlation—Hilbert transform—linear approximation,
(h) spectrum squared—cumulative sum—linear approximation,
(i) spectrum squared—cumulative sum—logistic approximation.

## 4. Experimental Data Analysis

The experiment was conducted using standard laboratory equipment. Measurements were performed in reflective mode with the following components: An Optical Spectrum Analyzer (OSA) AQ6370D (Yokogawa Electric Corporation, Kanagawa, Japan), a Superluminescent Diode S5FC1550P-A2 (SLD) (Thorlabs Inc., Newton, NJ, USA), an Optical Circulator 6015-3-APC (Thorlabs Inc., Newton, NJ, USA), and a Fibre Bragg Grating (FBG) used as a temperature sensor inscribed in the hydrogen loaded single-mode optical fibre using an excimer laser (Coherent Inc., Santa Clara, CA, USA). Light emitted from the SLD source was transmitted through an optical fibre to the first port of the optical circulator and subsequently directed to the second port, which was connected to the FBG sensor placed inside a temperature chamber. The reflection spectra were measured using the OSA, with a resolution of 0.04 nm, connected to the third port of the circulator.

**Figure 18:**
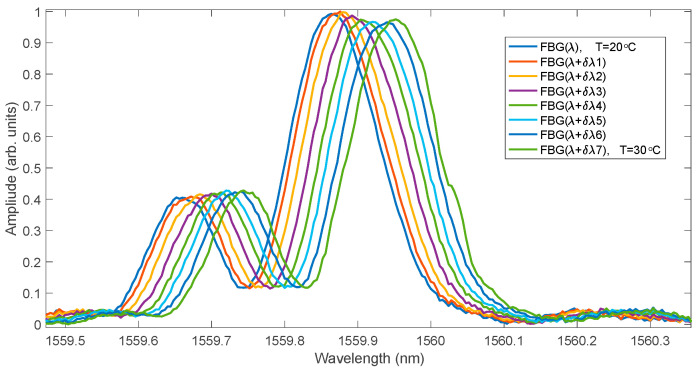
Experimental spectra of the FBG measured during heating in a thermal chamber.

**Figure 19:**
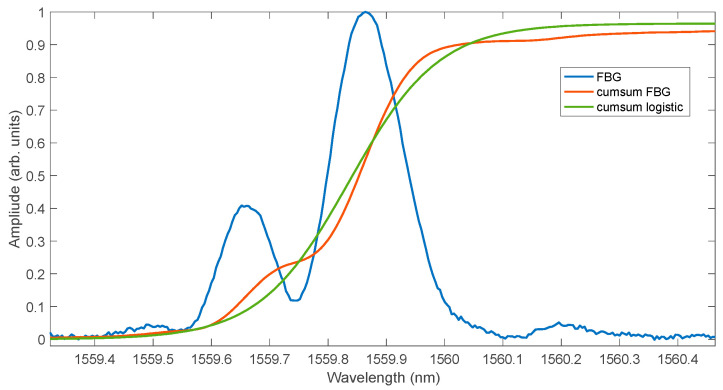
Approximation of the cumulative sum of the experimental spectrum using a logistic curve.

The spectra of the experimental grating for several measured temperatures are shown in Figure 18. The grating has a characteristic spectrum with a significant side lobe amplitude for a shorter wavelength than the main lobe. Figure 19 shows the normalised cumulative spectrum and its approximation by means of a logistic function.

**Figure 20:**
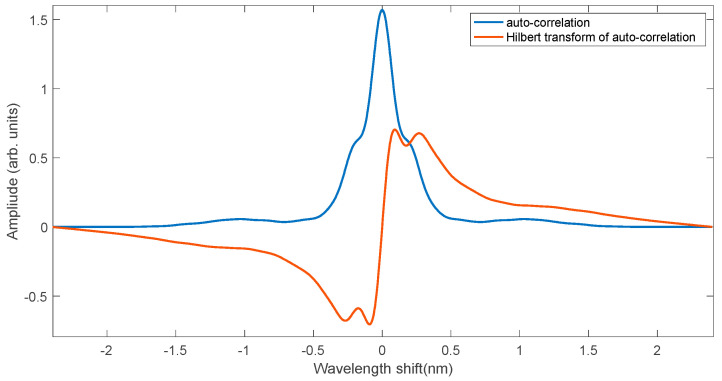
Autocorrelation of the first measured spectrum and its Hilbert transformation.

Figure 20 shows the shape of the autocorrelation function of the experimental grating spectrum and the Hilbert transformation of this function. Interestingly, the autocorrelation function is an even function, despite the lack of symmetry of the FBG spectrum itself. The Hilbert transformation is an odd function for which it is possible to determine the zero position. The method combining the cross-correlation functions and the Hilbert transform can be used to compare the Bragg wave shift values with the values calculated using the cumulative sum.

**Figure 21:**
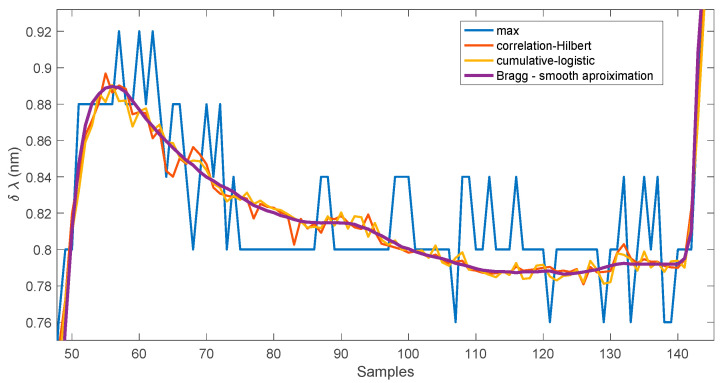
Bragg wavelength shift estimation results for experimental measurements.

During the experiment, the grating was placed in a heating chamber at a temperature of 20 °C and then the temperature was set to 30 °C. Figure 21 shows the course of the Bragg wavelength shift from the final moment of heating through the temperature stabilisation phase to the moment of setting the temperature in the heating chamber to 40 °C. The heating chamber maintains the temperature with an accuracy of 1 °C. The spectral resolution of the measurement was 0.04 nm, which can be seen by analysing the course of changes in the Bragg wavelength determined by the maximum value of the spectrum method. Individual changes in the shift value are much smaller for the other two methods. Analysing the courses of the correlation method and the cumulative sum method, it can be seen that there are some impulsive deviations from the smooth trend curve of the temperature change for both methods.

## 5. Conclusions

Using a simple method of summing successive spectral points, a new approach to determining the shift in the Bragg grating spectrum was proposed. The new spectrum transformation together with the linear function approximation algorithms and the logistic function demonstrate good properties for determining the wavelength shift. The results of the proposed method were compared with popular methods such as the cross-correlation algorithm and the Hilbert transform. Additional denoising performed using the wavelet transform does not change the properties of the proposed method. An additional reduction in the root mean square error can be obtained by squaring spectrum before calculating the cumulative sum and then performing the approximation with a logistic or linear curve. By calculating the difference between the cumulative sum calculated from the left and right side, the shape of the odd function with zero crossing can be obtained. This allows achieving the root mean square error for linear approximation at the same level as for the logistic function approximation. The numerical analyses carried out show that the proposed method can be used due to its simplicity and properties comparable to more complex methods.

## Data Availability

Data are contained within the article.
